# Fall Recognition Based on an IMU Wearable Device and Fall Verification through a Smart Speaker and the IoT

**DOI:** 10.3390/s23125472

**Published:** 2023-06-09

**Authors:** Hsin-Chang Lin, Ming-Jen Chen, Chao-Hsiung Lee, Lu-Chih Kung, Jung-Tang Huang

**Affiliations:** 1 Graduate Institute of Mechanical and Electrical Engineering, National Taipei University of Technology, Taipei City 10608, Taiwan; 2 Division of Nephrology, Department of Internal Medicine, MacKay Memorial Hospital, Taipei City 10449, Taiwan; 3 Department of Medicine, MacKay Medical College, New Taipei City 25245, Taiwan; 4 Department of Nursing, MacKay Junior College of Medicine, Nursing, and Management, Taipei City 11260, Taiwan; 5 Division of Gastroenterology and Hepatology, Department of Internal Medicine, MacKay Memorial Hospital, Taipei City 10449, Taiwan

**Keywords:** fall recognition, fall verification, smart speaker, Internet of Things

## Abstract

A fall is one of the most devastating events that aging people can experience. Fall-related physical injuries, hospital admission, or even mortality among the elderly are all critical health issues. As the population continues to age worldwide, there is an imperative need to develop fall detection systems. We propose a system for the recognition and verification of falls based on a chest-worn wearable device, which can be used for elderly health institutions or home care. The wearable device utilizes a built-in three-axis accelerometer and gyroscope in the nine-axis inertial sensor to determine the user’s postures, such as standing, sitting, and lying down. The resultant force was obtained by calculation with three-axis acceleration. Integration of three-axis acceleration and a three-axis gyroscope can obtain a pitch angle through the gradient descent algorithm. The height value was converted from a barometer. Integration of the pitch angle with the height value can determine the behavior state including sitting down, standing up, walking, lying down, and falling. In our study, we can clearly determine the direction of the fall. Acceleration changes during the fall can determine the force of the impact. Furthermore, with the IoT (Internet of Things) and smart speakers, we can verify whether the user has fallen by asking from smart speakers. In this study, posture determination is operated directly on the wearable device through the state machine. The ability to recognize and report a fall event in real-time can help to lessen the response time of a caregiver. The family members or care provider monitor, in real-time, the user’s current posture via a mobile device app or internet webpage. All collected data supports subsequent medical evaluation and further intervention.

## 1. Introduction

With an aging society, the healthcare of the older population has become a major public health concern all over the world. The rapid development of information technology in recent years provides an ideal solution to improve the quality of life of the elderly. Thus, there is an urgent need to develop an efficient fall detection system to prevent the occurrence of unexpected adverse events. Fall events are the second leading cause of unintentional injury and death all over the world [1]. It is estimated that there are 684,000 individuals who die from fall events each year [2]. People older than 65 years old are more susceptible to a lethal fall or other adverse events [2]. Fall events will incur significant medical expenses, and the patients will need long-term rehabilitation therapy. Such incidents most frequently occur in aging people due to musculoskeletal disorders [3,4,5]. A variety of neurological disorders may lead to falls. For example, patients with dementia are at high risk of a fall [6,7]. Patients with Parkinson’s disease (PD) often have gait disturbance [8,9,10]. Elderly people with cardiovascular disorders are also at high risk of a fall [11,12,13].

Elderly people requiring care during the entire day usually depend on the availability of their family members to provide such assistance. However, family members might not provide appropriate help, especially in an emergency situation. Aging people living alone are at high risk of falls [14]. Therefore, long-term healthcare (LTC) has become increasingly important. Existing types of care institutions are mainly divided into home-based and community-based. With the rapid growth of LTC services, it is not easy to find qualified caregivers [15]. The lack of caregivers in nursing facilities may result in aging people being neglected or not receiving comprehensive care. Compared with nursing centers, most elderly people still prefer home care. However, their family members need to go out to work and cannot take care of them at home for a long time. At this time, the importance of home care increases a lot, and fall monitoring will be one of the key points of an at-home care system [16]. In addition to the immediate injury caused by falls, the mortality rate of elderly people who have fallen is significantly higher than elderly people who have not suffered any falls [17], and the subsequent related medical costs are also quite high [18]. Therefore, it is urgently required to develop a fall detection system for the elderly to minimize their risks of a fall.

Smart speakers show promise in enhancing the aging population’s well-being. They provide great help for the elderly with a chance to have more connection with other people, as well as health-related information and services. They pose great value for older people to assist with daily life, especially for those who are living alone.

We propose a system for the recognition of falls based on a chest-worn wearable device and verify falls through a smart speaker and Internet of Things (IoT) technology. The wearable device is embedded with a nine-axis inertial measurement unit (IMU) sensor, including a three-axis accelerometer, gyroscope, and magnetometer. It can determine the behavior state including sitting down, standing up, walking, and falling. We can also determine the direction of the fall, such as falling forward, falling backward, falling to the left, and falling to the right. Forward falls are the most frequent cause of upper limb fractures [19].

Fall-related head injuries account for 60% of hospital admission [20,21,22,23,24] and more than 50% of deaths [25,26] in aging people. Therefore, the development of an efficient solution to prevent fall-related head injuries is an urgent and crucial issue. The number of studies focused on fall detection has increased dramatically over the previous ten or more years. However, most of the studies only aim at identifying if the falling events occur. These solutions neglected the direction of a fall and the degree of physical injury related to a fall. The degree and extent of physical injury are related to the direction of a fall. A backward fall increased the risk of hip or pelvis injury. Forward falls increased the risk of impact to the hand or wrist [27]. Head injuries were common when elderly people fall, especially forward falls compared with the other directions of a fall event [28]. Different directions of a fall might lead to different degrees of injury to the fallen person. We propose a fall detection solution that can reliably and accurately recognize the different direction of a fall. Therefore, our fall detection system also provides the classification of the risk of falls. This could provide timely and practical fall-related information to the doctors to evaluate the probable location and extent of the head injury, especially for fatal intracranial hemorrhage.

During the fall, the impact force can be determined by the calculation of acceleration changes. In addition, with the aid of the IoT and smart speakers, we can confirm whether the user has fallen by asking from smart speakers. Therefore, the contributions of this research are as follows:

(1) We develop a chest-worn wearable device with the integrated operation of an IMU sensor and a precision barometric altimeter.
(2) Based on the chest-worn device, we can determine the direction of the fall, such as falling forward, falling backward, falling to the left, or falling to the right.
(3) We also can determine the intensity of a fall generated from the various behavior states such as standing, sitting, and walking.
(4) With the aid of smart speakers and the IoT, false alarms can be reduced by directly asking the fallen person for further confirmation.
(5) We provide a smart wristband for sharing important physiological information to the chest-worn device and cloud every second to know if the health condition gets worse after a fall event.

## 2. Related Work

In order to minimize the risk of physical injury and hospitalization caused by a fall, an efficient fall detection system is urgently needed. Many studies have been dedicated to investigating fall detection using a great variety of sensing methods. These fall detection systems can be classified into several categories using different sensors, such as wearable sensors, vision-based sensors, and environment sensors. However, the performance of these studies is still not satisfying due to some drawbacks and limitations. Auvinet et al. introduced a fall detection system with multiple cameras using an occlusion-resistant method. However, the setup of the system is complicated, and the accuracy is low if there are two or more people in the same room [29]. Feng et al. developed a fall detection system based on a pressure sensor to measure the changes in body weight on the ground. However, it is difficult to distinguish the human body from other objects, such as animals, in the system [30]. Maheshwari et al. designed a fall detection system, but the detection will fail if there are multiple persons in the same space during the fall event [31]. Li et al. proposed a fall detection system utilizing acoustic sensors to measure the sound of a fall. However, the accuracy of detection by an acoustic array diminishes if the person is more than 5 m away [32].

Over the previous ten or more years, the development of fall detection systems became a quite hot topic due to the aging society. Various technologies and methodologies have been adopted, such as the IoT [33,34,35] and artificial intelligence [36,37].

The fall detection system based on wearable sensors has become more popular over the previous decades because of its compact size, low energy consumption, and inexpensiveness. There are various studies investigating fall detection based on a wearable sensor with a built-in accelerometer. However, they are lacking an alarm-sending system to call for help from the fallen person [38,39,40,41,42]. Several studies provide a designed solution for early fall detection, but they do not incorporate the direction of fall measurement in their design [43,44,45,46].

Vision-based sensors are affected by the number and location of cameras. The method can only play a role in a fixed area due to the limited number of cameras, and their locations are fixed. In addition, the privacy invasion of the users is a major concern. Environment-based sensors are easier to have false alarms because any change in the environment, such as falling objects in the room, heavily influences the performance of sensors and their accuracy [47].

## 3. Development Environment and Method

### 3.1. Development Environment of Falling Recognition

**Figure 1:**
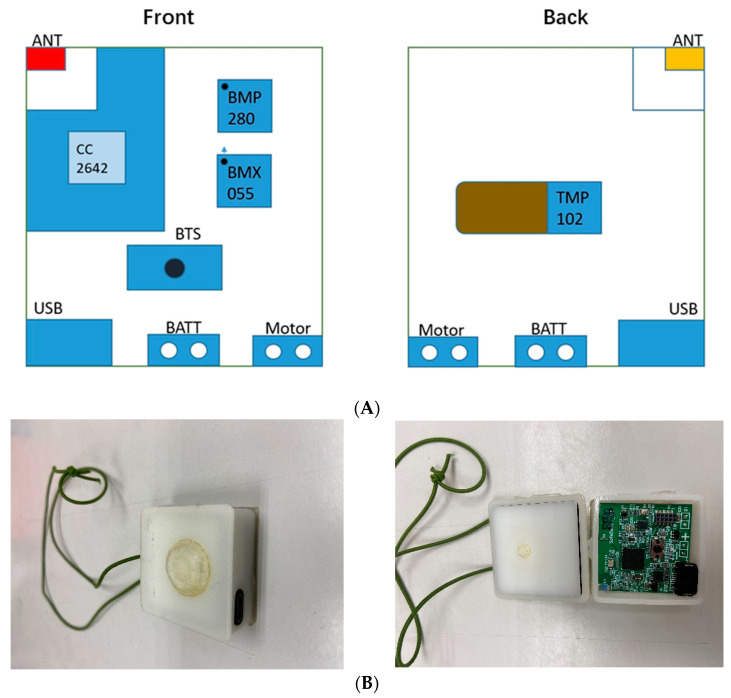
(**A**) The circuit board layout of the chest-worn wearable device. (**B**) The appearance of the chest-worn wearable device.

In our research, a configuration diagram of each component of the circuit board layout of the chest-worn wearable device is shown in Figure 1. The Bluetooth 5.2 communication module is composed of an SOC (TICC2642, Texas Instruments, Dallas, TX, USA), a ceramic antenna, Button Tactile Switch (BTS, Jameco ValuePro, Taipei, Taiwan), a micro-USB charging connector, a vibration motor, a nine-axis IMU (inertial measurement unit) sensor (BMX055, Bosch Sensortec, Reutlingen, Germany), a barometer sensor (BMP280, Bosch Sensortec, Reutlingen, Germany), and a 150 mAh Li-ion battery. The nine-axis IMU sensor consists of a three-axis accelerometer, a three-axis gyroscope, and a three-axis magnetometer. The acceleration, angular velocity of rotation, and geomagnetic direction can be measured, respectively. The sampling rate of the accelerometer is set at 100 Hz, and the measurement range is ±4 g. The wearable device can recognize the user’s posture and body rotation. Thus, it can determine the current behavior state such as sitting, standing, walking, lying down, and falling. The user can press the BTS to announce the emergency situation through Bluetooth at any time. If the cloud has received the emergency signal, the vibration motor will vibrate so that the patient can wait for the rescuer to come. It is based on the rotation angle and moving distance of the wearable device. In order to accurately recognize standing, sitting, and identify the posture before falling, an accurate barometer sensor (BMP280) is selected. In the following section, it can be shown that using a barometric altimeter can improve the accuracy of fall determination, especially the behavioral state before a fall. Indoor positioning can be performed by dead reckoning navigation [48] without using fixed tags such as pressure-sensing cushions, pedal mats, or magnetic reed switches [49,50].

**Figure 2:**
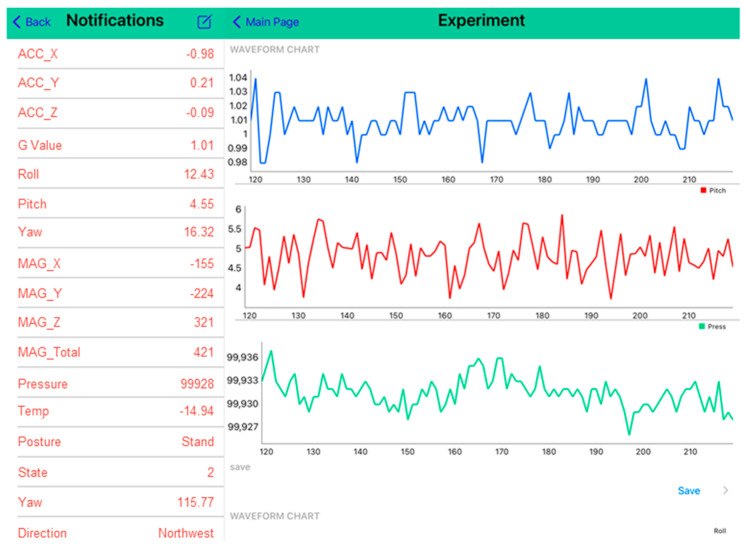
Real-time monitoring and data collection in the iOS tablet app.

Figure 2 shows real-time monitoring and data collection on our self-developed iOS app. The chart on the left displays the available data, allowing the caregiver to monitor the user’s current status with this chest-worn wearable device, such as his/her posture and position. The chart on the right shows nearly 100 items of data. The data can be stored in sections or continuously, allowing the caregiver to view the data changes of the user in a short period of time. The caregiver can also access historical data through a data storage function.

### 3.2. Development Environment of Falling Verification

The development environment of falling verification in this study consists of a variety of hardware.

**Figure 3:**
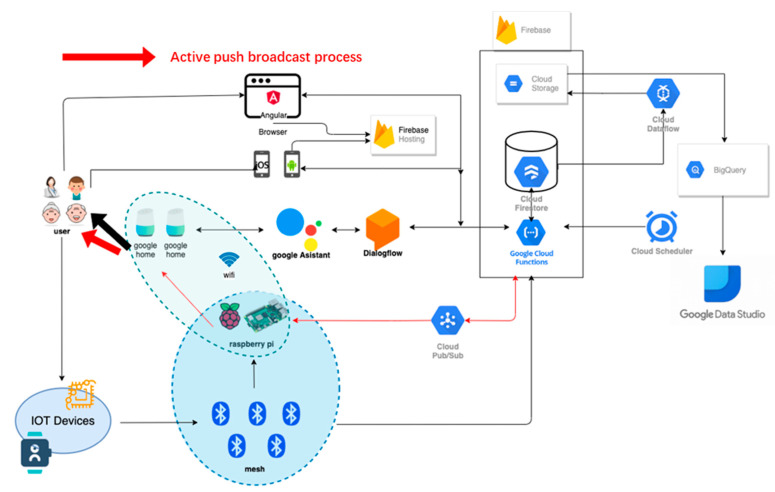
Falling verification and the IoT care structure.

(1) bNode modules:

As shown in Figure 3, network deployment in large areas, such as factories or nursing centers, requires a mesh function [49]. Thus, the advantage of dual Bluetooth SOC chips is used to realize this function. The dual chips could play a central and peripheral role. The former is responsible for broadcasting and sending data and the latter is in charge of scanning and receiving data. Both communicate with each other and transmit data through the UART communication interface, thereby accelerating the speed of data processing and transmission. However, the communication range of BLE 5.0 is longer in small areas, such as a home environment, and data can be transmitted to bwRouter without the mesh function.

(2) Smart wristband:

It is used to record heart rate, calories consumed, the number of steps, and upload signals with a fixed frequency. For the most part, the smart wristband is able to provide the above crucial physiological information to the chest-worn device and cloud continuously to check whether the health condition of the user gets worse after a fall.

(3) Chest-worn IMU sensors:

A variety of sensors set in the IMU and barometer processed by a BLE5.0 SOC can perform the monitoring of the user’s posture, movement, height, fall event, and indoor positioning. The daily activity pattern of the elderly can be recorded. Therefore, in a home environment, the single chip of the BLE5.0 SOC is responsible for broadcasting to the bwRouter and scanning signal from the smart wristband, that is, Multi Role, but the speed of data transmission will be reduced.

(4) Raspberry Pi:

Raspberry Pi acts as the bwRouter of the system and actively pushes speaker programming in the same domain. This allows it to act as the hub of various sensor values uploaded to the cloud and the server of the active pushing of messages.

(5) Google Home nest mini:

It is a hands-free speaker from Google. In this study, the user can ask Google to get the message. The conversation logs, posture recognition, physiological data, and location of the user can be collected.

Then, this information is transmitted to the cloud through the Bluetooth mesh network. A variety of cloud services can be used.

Firebase Firestore:

Responsible for storing various sensor information and conversation logs.

Firebase hosting:

Responsible for hosting web pages.

Firebase storage:

Stores the data output from the dataflow.

Firebase functions:

Deploys various programs to run or trigger in the cloud 24 h a day.

Firebase authentication:

Responsible for managing web user accounts.

Cloud scheduler:

Programs can be triggered periodically or at a specific time by setting parameters.

Pub/Sub:

Responsible for the delivery of conversation messages.

Dataflow:

Outputs Firestore data to the specified area.

BigQuery:

Data can be organized and appended to overwrite the results in a table through SQL queries.

Datastudio:

Visualizes the data and forms various kinds of tables and charts.

Dialogflow:

A natural language processing platform that can design all kinds of dialogues.

A natural language understanding platform that makes it easy to design and integrate a conversational user interface into mobile apps and web applications.

Actions on Google:

A platform for setting up care assistants on smart speakers.

Figure 3 shows the architecture of the hardware and services that are constituted by the network, cloud, and smart speakers. Users can monitor the health status of aging people through a variety of interfaces such as internet webpages, mobile device apps, smart speakers, or smart wristbands. The web and mobile sides are deployed in Firebase hosting. Users can set up devices, query their status, and input data to store in the Firebase cloud database. At the same time, IoT devices, including smart wristbands, continuously collect physiological data, height value, and acceleration through BLE and edge devices. These data are uploaded and stored to Firebase through functions. In addition, the most important interface in this study, the smart speaker with voice interaction, plays a vital role in cloud architecture. The new interaction medium provides both active reminders and passive questioning modes so that the elderly can easily obtain the necessary information and health advice through communication. It provides great support for the elderly, with the benefit of a hands-free interaction mode to deal with any requests. Passive questioning is performed through the Google Assistant service developed in this study. The Dialogflow-designed dialogues and webhooks can obtain physiological information and record user conversations to the Firestore. Whenever the system detects a fall event, rapid heart rate, or wristbands not being worn, these messages will be transmitted via Pub/Sub to the Raspberry Pi within the same network domain as the smart speaker. Then, messages are sent to the specific smart speaker through the internal running program to achieve active pushing of information to the user. Finally, the data in the Firestore is output into JSON files to storage through dataflow. After data processing, BigQuery can organize the data to form tabular data through the SQL syntax and generate reports through Data Studio.

### 3.3. Active Push Broadcast

**Figure 4:**
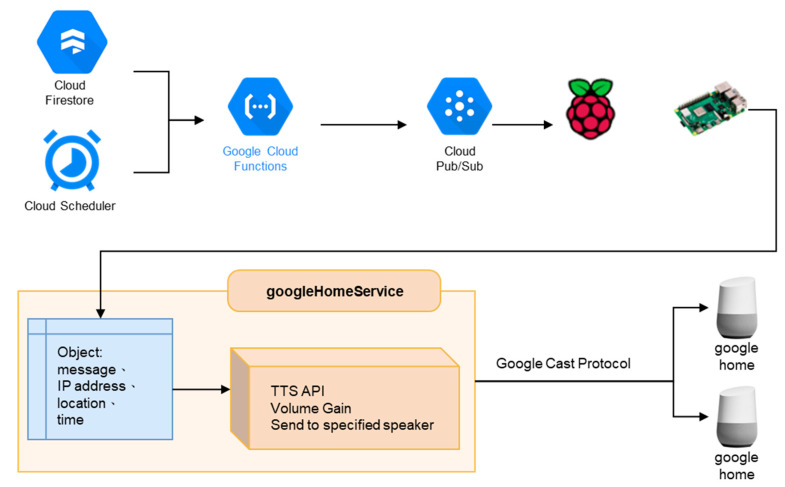
Active push broadcast structure.

In order to broadcast important information in real-time, we deployed individual programs in Firebase functions. A timer is set by their triggering methods. After being triggered two time, the objects are sent through Cloud Pub/Sub and pushed to the Raspberry Pi to receive the program running in the message field of the object. The message field in the object records the voice to be sent through the Google TTS API. The voice is converted into a voice message. The nearest speaker to the user is determined, and then it notifies the important message to the user through Google Cast Protocol, as shown in Figure 4.

In this study, the active push messages include regular reminders to wear the wristband, reminders of sitting for more than an hour, abnormal physiological data, and fall notifications.

The technical specifications of the sensors used for fall detection, including the accelerometer, gyroscope, magnetometer, and barometer, are shown in Table 1.

### 3.4. Methods

In our system, a data analysis of the sensors in the chest-worn wearable device is performed via the Madgwick algorithm (gradient descent algorithm). Using the Madgwick algorithm, the altitude calculated by the accelerometer and magnetometer is linearly fused with the attitude obtained by the integration of the gyroscope. Finally, the three-axis rotation angle of the roll, pitch, and yaw with high precision is obtained. The resultant G value in Equation (1) and the pitch are calculated by the square root of the three-axis acceleration, and the Kalman filter is performed on the relative altitude change detected by the barometer. Altitude change is observed every 1.5 s to assist in determining the behavior state of sitting down and standing up.

(1) $$G_{value}=\sqrt{{ACC_{x}}^{2}+{ACC_{y}}^{2}+{ACC_{z}}^{2}}$$

**Figure 5:**
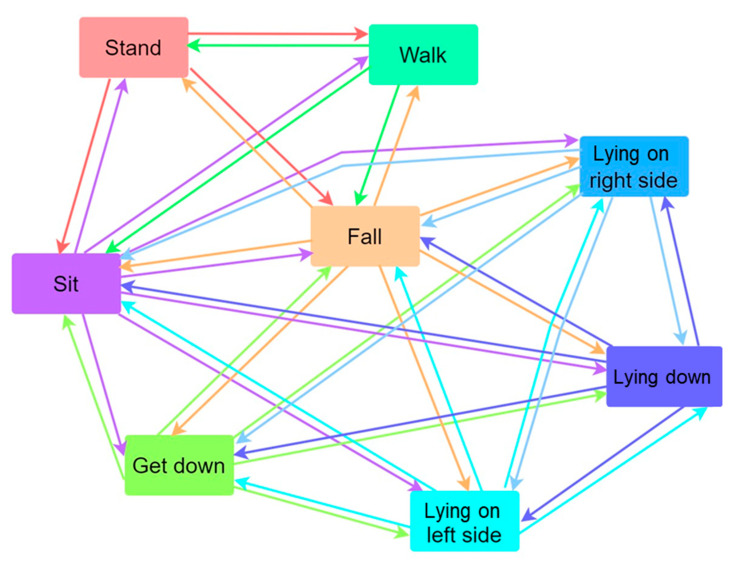
Behavior state switching using the FSM.

**Figure 6:**
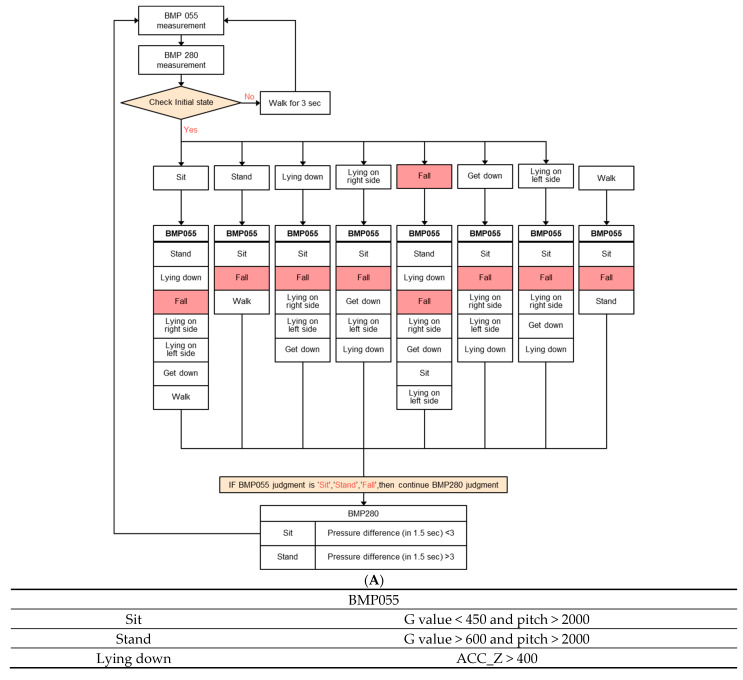

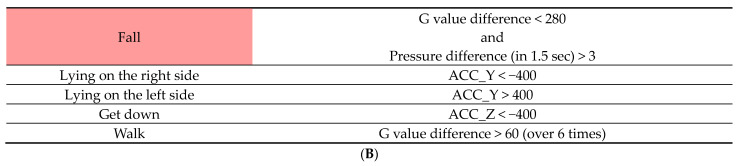
(**A**) The software flow diagram of the system while the FSM is executed. (**B**) The parameter of eight kinds of behavior states.

We use a finite-state machine (FSM) to switch behavior states and execute corresponding events according to the current behavior state, as shown in Figure 5 and Figure 6. An FSM is an efficient way to organize and manage the program state. The server can perform corresponding logical processing according to different states or message types, making the program logic clear and easy to understand. The FSM is divided into the Moore state machine and the Mealy state machine. The output of the former is only determined by the current state, and the latter generates output according to the current state and input. Since human behavior is changeable and some behaviors are unpredictable, the Mealy state machine is used here to design the program to determine how to switch the behavior state. We determine the next state by comparing the input state with the current state, executing the corresponding function action and jumping to the next state. As shown in Figure 2, when the wearer puts on the device and stands, the next behavioral state may be to stand up or sit down. If the next behavior is standing, execute the stand-up function and jump from the current state to the standing state. If the current state is standing and the input state is sitting, the state machine does not match, and the corresponding sitting function will not be executed, nor will it jump to the sitting state. Behavior restrictions are implemented in this way to reduce the possibility of misjudgment. Therefore, when first wearing the device, the wearer needs to get up and walk ten steps to make the behavioral state consistent with reality before recording the experiment.

### 3.5. The Principle of Fall Detection

The acceleration while falling will change drastically, which is significantly different from when standing or sitting. Therefore, the determination of whether a fall has occurred uses the vector sum of the accelerometer and barometer in the IMU. It is based on the change in the G value. If the change in the G value is greater than 600 and the change in air pressure is greater than 3, it will be determined as a fall.

### 3.6. Experimental Method

Simulated falls were performed in the following three situations. (1) A person is standing and then falls forward, backward, to the left, and to the right. (2) Standing up while sitting on a chair, and then falling forward, backward, to the left, and to the right. (3) Sitting on a chair, and then falling forward, backward, to the left, and to the right.

Fall recognition is determined by the G value in the weightless state and the results of measurement in the barometer. Fall verification was performed to confirm whether the device has detected a fall. There are twelve test participants, including seven young subjects (22–25 years old) and five middle-aged subjects (36–60 years old).

### 3.7. Experiment Procedure

The system test contains four kinds of fallings including forward, backward, leftward, and rightward.

(A) Falling backward:

(1) The subject stands still with their back to the cushion, (2) walks 10 steps in place, and then lies back on the cushion. (3) The subject gets up from a squatting posture.

(B) Falling forward:

(1) The subject stands still 1.5 to 2 m in front of the cushion, (2) walks two steps forward, and then lies on the cushion. (3) The subject gets up from a squatting posture.

(C) Falling leftward and rightward:

(1) The subject stands still 1.5 to 2 m on the side of the cushion, (2) moves sideways, and then lies on their side on the cushion. (3) The subject gets up from a squatting posture.

After wearing the chest device, the user needs to walk for about three seconds to initialize a state, as shown in Figure 6A. Then, the FSM is executed according to the behavior state chart in Figure 5 and the parameter of various states in Figure 6B. The G value represents the sum of the squares of the XYZ three-axis acceleration of the IMU (ACC_X, ACC_Y, and ACC_Z). The pitch is the angle at which the user’s upper body is pitched. The pressure difference is the change in the barometer’s measured value. After the initial state check is completed, there can be eight kinds of states, as shown in Figure 6A. For example, after the sitting state is completed, there can be seven states, and the remaining states can be deduced, as shown in Figure 6B.

## 4. The Experiment of Recognition of Falls for Various Postures

The nine-axis accelerometer sensor and the barometer sensor in the wearable device are used to determine the current posture of the person. The behavior state is determined by the switching of the state machine. When the wearable device is powered on, the state machine of the IMU is in the initial state. If the user sits down, the state machine switches to the sit-down mode and makes a judgment on the behavior state and state machine. After data analysis, the moving path and distance of the user can be obtained, and whether the amount of physical activity of the user is sufficient can also be checked. For the elderly with dementia, who may wander away from home or even get lost, the cloud can analyze the moving path, and the system can send a notification to the caregiver right away. If a person is suddenly subjected to a violent change in the resultant force of the accelerometer, the system can also inform the caregiver that the person may be at high risk of a fall. All the above behavioral patterns are stored in the cloud database so that caregivers or doctors can identify any early signs of health issues in advance. In order to detect the user’s movements, it takes 0.1 s to read the IMU and altimeter to determine if a fall has occurred.

### 4.1. Recognition of Falls

In the study, we conducted experiments to verify if the wearable device can recognize the fall in a different direction for various postures, including sitting, standing, and standing up. Determination of a fall is based on the reduction in the resultant G value. The weightlessness of a fall is more than sitting down, and the peak value after weightlessness and impact force experienced by the user are also larger than sitting down. The changes in roll and pitch angles can determine the direction of the fall. If the body leans to the right, the roll angle is positive, otherwise, it is negative. When the body leans forward, the pitch angle is positive, otherwise, it is negative.

### 4.2. Recognition of a Fall While Sitting

**Figure 7:**
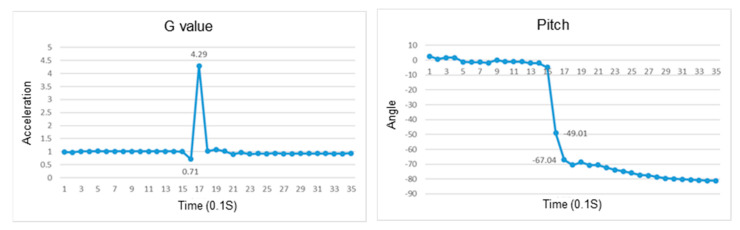
The change in the resultant force and pitch angle of falling backward while sitting on a chair.

**Figure 8:**
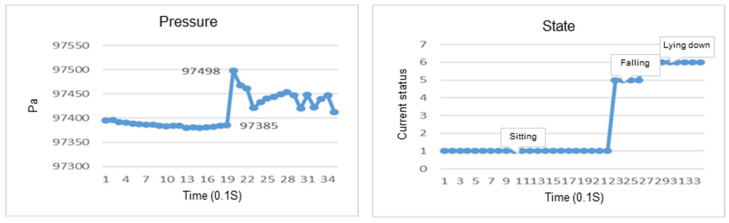
The change in the atmospheric pressure and state of falling forward while sitting on a chair.

**Figure 9:**
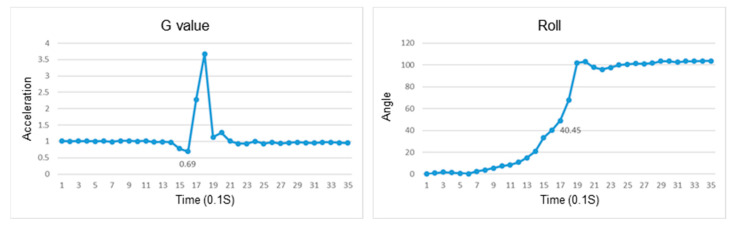
The change in the resultant force and roll angle of falling to the right while sitting on a chair.

**Figure 10:**
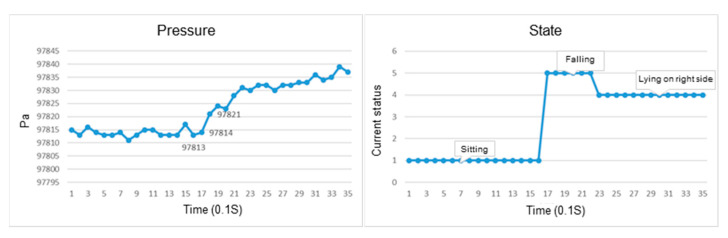
The change in the atmospheric pressure and state of falling to the left while sitting on a chair.

This section discusses the experiments on fall recognition in different directions, including facing forward, backward, to the left, and to the right while sitting on a chair, as shown in Figure 7, Figure 8, Figure 9 and Figure 10. The user experiences a fall from sitting to standing. The wearable device can determine the fall event.

### 4.3. Recognition of a Fall While Standing

**Figure 11:**
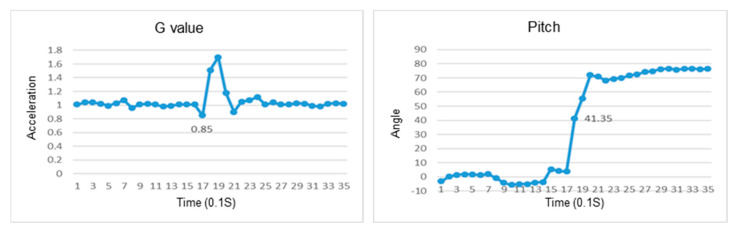
The change in the resultant force and pitch angle of falling forward while standing.

**Figure 12:**
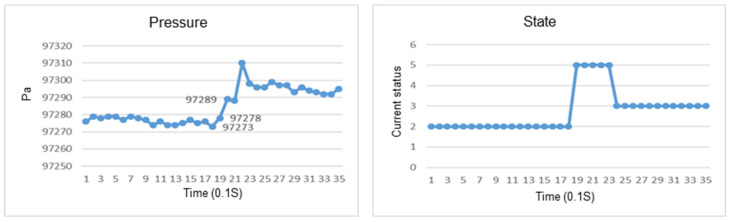
The change in the atmospheric pressure and state of falling backward while standing.

**Figure 13:**
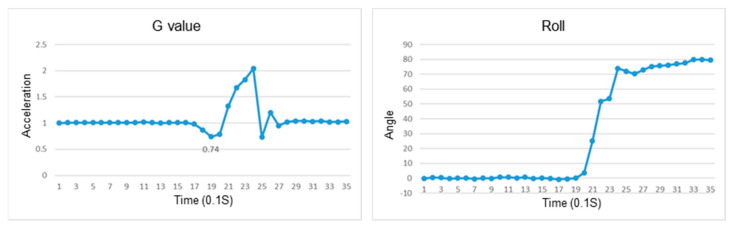
The change in the resultant force and roll angle of falling to the right while standing.

**Figure 14:**
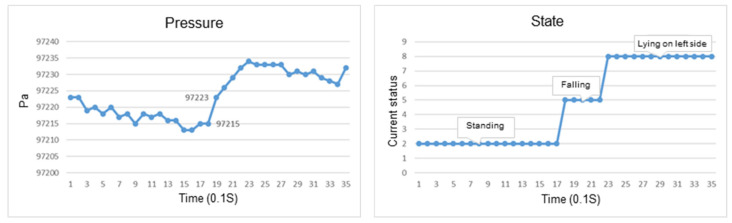
The change in atmospheric pressure and state of falling to the left while standing.

This section discusses the experiments on fall recognition in different directions, including facing forward, backward, to the left, and to the right while standing, as shown in Figure 11, Figure 12, Figure 13 and Figure 14. The user experiences a fall while standing. The wearable device can determine the fall event.

### 4.4. Recognition of a Fall While Standing Up

**Figure 15:**
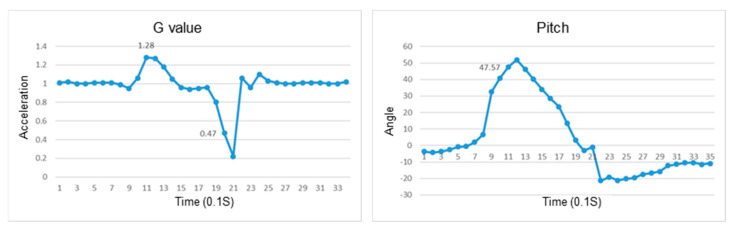
The change in the resultant force and pitch angle of falling backward while standing up.

**Figure 16:**
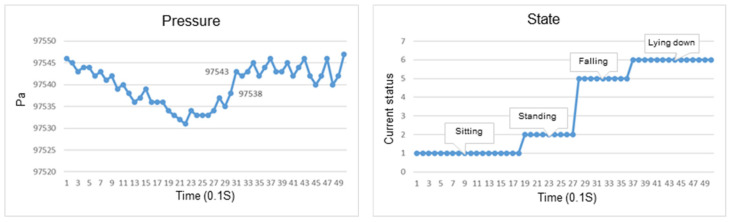
The change in the atmospheric pressure and state of falling forward while standing up.

**Figure 17:**
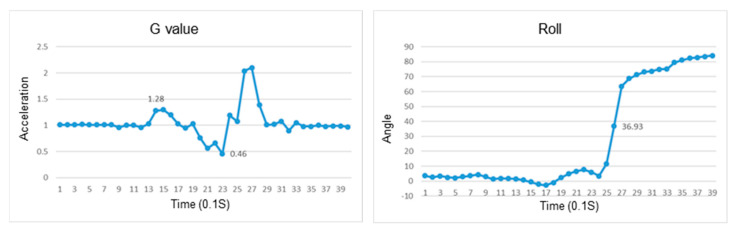
The change in the resultant force and roll angle of falling to the right while standing up.

**Figure 18:**
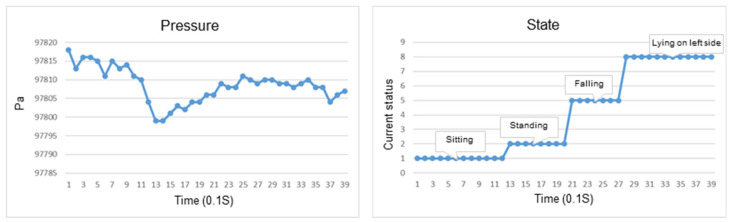
The change in the atmospheric pressure and state of falling to the left while standing up.

This section discusses the experiments on fall recognition in different directions, including facing forward, backward, to the left, and to the right while standing up, as shown in Figure 15, Figure 16, Figure 17 and Figure 18. The user experiences a fall while standing up. The wearable device can determine the fall event.

### 4.5. Falling Verification

**Figure 19:**
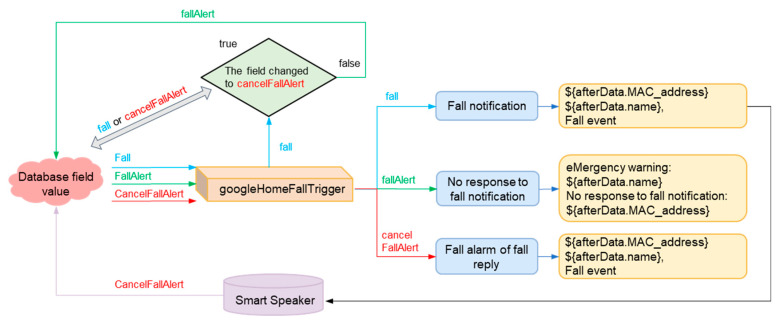
Fall push casting process.

Verification of a fall mainly relies on Firestore to record the fall status field through the Google-HomeFallTrigger deployed in the function and decides whether the care assistant responded to the false alarm as the basis for sending various messages, as shown in Figure 19. The function Google-HomeFallTrigger sends a message to the smart speaker and sets a timer to confirm whether the value of this field is still falling after 30 s. If so, it is necessary to update FallAlert to send messages to the smart speakers again. Users can also cancel the alarm within 30 s after pushing the alarm by asking Google Home. False alarms of falls can be reduced by the above two methods. With the two-stage fall notification, the caregiver can immediately judge the situation and reach the user in time.

**Figure 20:**
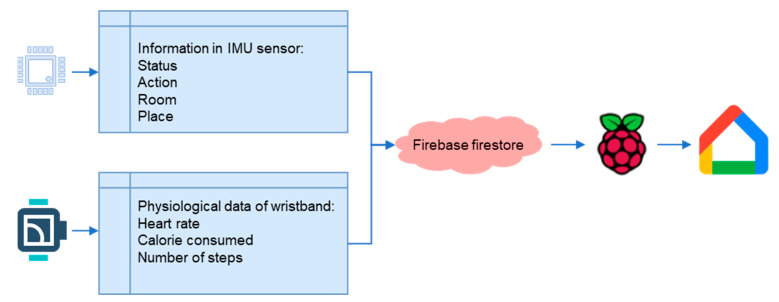
Physiological signal pushing process after a fall event.

In addition, fall-related data is also stored in the cloud database, including the physiological data obtained from the wristband, posture recognition, behavior state, and fall location from the IMU sensor. When the physiological information of the elderly is abnormal, the system will send a message to the Google speaker through an active push broadcast to notify the caregiver. The physiological data of the elderly after a fall can be provided to the doctor for evaluation and diagnosis as the basis for further treatment and long-term healthcare, as shown in Figure 20.

The sensitivity, specificity, and accuracy of the proposed solution include the following three categories: a fall while sitting down, a fall while standing, and a fall while standing up. The fall algorithm achieved 0.94, 0.96, and 0.95 on sensitivity, 0.95, 0.97, and 0.95 on specificity, and 0.95, 0.96, and 0.95 on accuracy in categories A, B, and C, respectively, as shown in Table 2.

## 5. Discussion

With the global population aging gradually, enhancing the physical health and quality of life of the elderly represents a fundamental issue. Due to the soaring need for assisting in the healthcare of aging people, it is quite urgent to have enough qualified caring manpower. Falls are a major cause of unintentional death and injury. The risk of a fall and fall-related health disorder increases with age. It is remarkably high for older people, especially if they suffer from a chronic disease. Therefore, an efficient solution for early fall detection is a key factor to ensuring healthy aging life.

The utilization of mobile phones can certainly provide a function for the alarm. However, the mobile phone does not have the function of turning on the monitoring at any time, such as the smart speaker. For elderly people in the home environment, they are not used to carrying their mobile phones with them. The volume of the mobile phone is generally not very loud and does not have a good effect at long distances. Mobile phones could be connected to the same source of information as smart speakers, in which case they would have pretty much the same programming and information. However, a typical smart speaker has certain “mechanical advantages”. For one, it probably has a better speaker. Additionally, many smart speakers have multiple microphones, and perhaps better microphones, which most phones do not, so the speaker can use the differences between what the microphones pick up to tell what direction your voice is coming from. Smart speakers, such as Amazon Echo and Google Home, are always listening. You can cook and have food in your hands and easily set a timer without waking up your phone or getting it out. You can walk into your house and turn on your preferred lights without getting the phone. Almost any sort of home automation works much better with Amazon Echo than with a phone. As others have said, the sound is better from such a device than from your phone. Mobile phones have poor speakers compared to smart speakers. You can fill a reasonably sized room with decent-sounding music from the smart speaker. They are also a lot better at recognizing your voice from a distance, even at a normal volume if it is playing music.

A wrist-worn device has the advantage of lower invasiveness. The wrist, allowing more mobility, frequently serves as a position for sensors embedded in wearable devices [51]. However, the positions with reduced movability on the body are more suitable for some applications related to the body’s center of mass. For example, IMUs on the chest provide some advantages compared with other positions in the body for systems of fall detection and activity recognition. Because the chest is near the center of mass in the human body, it is an appropriate site for the systems that aim to detect a fall and classify physical activities. Chest-worn IMUs are used in applications such as activity reorganization [52,53] and fall detection [54]. Zhang et al. compared three positions including the wrist, chest, and waist for physical activity measurement. The participants concluded that the chest is preferred compared to waist for the test [55]. Some applications used IMU sensors attached to the chest using elastic straps [56,57] and stretching straps [58]. Compared to the above, the attachment of the IMU over the skin using adhesive tape might be as uncomfortable as elastic straps or stretching straps. However, it is still crucial to develop more user-friendly chest-worn inertial sensors in the future.

The chest-worn sensor can be used to recognize body movements at the same time, as shown in the flowchart of behavior state determination in Figure 5. Therefore, we can know the behavior state before a fall, such as standing, sitting, walking, or falling while sitting and standing. In addition, the direction and impact force of the fall are also available. It is very helpful to provide this important information to the doctor for the patients seeking medical care after a fall. The medical specialist can narrow the scope of the injury so as to quickly manage the injured area.

Parkinson’s disease (PD) is a neurodegenerative disease affecting more than six million patients all over the world. Sense4Care STAT-ON is a device, worn on the waist, based on a single wearable system that can monitor the movement patterns of patients with PD. Health professionals can obtain important physiological information for better decisions on the treatment of PD. The sensor has a battery life of 7 days for continuous operation (8 h per day). In our fall detection system, the IMU sensor is multifunctional and is able to recognize the direction, impact force of the fall, and posture before the fall. Therefore, the sampling rate of both the IMU and the barometer must be set at 100 Hz. As a result, the power consumption will be higher. The battery life is 24 h

The fall detection system based on wearable sensors has become more popular over the previous decades because of advantages such as its compact size, low energy consumption, and inexpensiveness. It was classified into threshold-based and machine-learning-based. The threshold-based system uses the fusion of data from multiple sensors to detect a fall. Any measured value outside the threshold means the occurrence of an abnormal situation. Wu et al. propose a fall detection solution with a single accelerometer-based wearable device [59]. The processing algorithm is based on the quaternion rotation angle and threshold of sum acceleration. The system has a compact hardware design with low power consumption. The sensor is located around the waist, which is very close to the center of the human body. The waist had been shown to be corresponding to body movement; thus, it is an appropriate site to mount the sensor in the fall detection experiment. However, only three participants were enrolled in this experiment. Adequate numbers of experimenters from different age groups could lead to higher reliability. Mao et al. proposed a fall detection system based on the sum acceleration and Euler angle [60]. A mobile phone is used to detect the fall event, and a fall alarm signal can be sent to the caregiver or the family. They claim that the location of the sensors influences the accuracy of fall detection. In their test, the location of the placement of the sensor includes the waist, shoulder, and foot of the participants. They concluded that sensor placement on the waist had the highest specificity compared to the shoulder and foot. Different fall directions might result in different degrees and extents of physical injury. They do not discuss the direction and impact force of the fall. In our system, we can detect a fall and verify whether it occurs. In addition, we are also able to determine the direction and intensity of the fall, which could be very crucial information for the doctor to evaluate and manage. Table 3 shows a comparison of existing solutions based on wearable sensors with our work.

There are several commercial solutions for fall detection and real-time alarms. A Lifeline AutoAlert system is designed to detect a fall event and gets access to help with two-way communications via pressing a call button. An Apple Watch is a wrist-worn wearable device with a built-in accelerometer and gyroscope sensors that can detect a fall. It also provides communication between the user and caregiver or medical staff. It does not include impact force and direction associated with the fall. A comparison of existing commercial solutions for fall detection and real-time alerting with our proposed system is shown in Table 4.

Our system can accurately recognize various types of body movement and user orientation through the nine-axis sensor and barometer fixed on the chest. In addition, the behavior state before the fall can be known by the FSM and experiments. The chest-worn sensor can be used to recognize body movements at the same time, as shown in the flowchart of behavior state determination in Figure 5. Therefore, we can know the behavior state before a fall, such as standing, sitting, walking, or falling while sitting and standing. In addition, the direction and impact force of the fall are also available. It is very helpful to provide this important information to the doctor for the patients seeking medical care after a fall. The medical specialist can narrow the scope of the injury so as to quickly manage the injured area.

Our research has some limitations, as shown below:

(1) In order to ensure accurate fall detection, the wearable device is attached to the user’s skin near the sternum of the chest with the adhesive. Medical adhesives are widely used in the medical field. Although the adhesive of the device is a medical type, it is still possible to be allergic to the materials in these adhesives. Thus, it is necessary to select a suitable adhesive individually.
(2) The wearable device uses a 150 mAH lithium-ion rechargeable battery, which needs to be charged every day. In order to ensure fall detection is performed 24 h a day, it is necessary to provide two chest-worn wearable devices, which can be used alternatively while the battery is charging.

## 6. Conclusions

We propose a smart healthcare system for fall recognition and confirmation based on a chest-worn wearable device and a smart speaker/IoT for the elderly. The recognition of a fall is based on accelerometer and gyroscope data in the chest-worn wearable device. Our system consists of a posture recognition model. The wearable device uses the three-axis accelerometer and gyroscope in the nine-axis inertial sensor to recognize the various postures of the user, including standing, sitting, and lying down. The resultant force was obtained based on the three-axis acceleration. The pitch angle was obtained by the three-axis acceleration and three-axis gyroscope through the gradient descent algorithm. Integration of the pitch angle and height value converted by the barometer can determine the behavior state such as sitting down, standing up, walking, and a fall event. Our smart system can clearly determine the direction of the fall, such as falling forward, falling backward, falling to the left, and falling to the right. The acceleration change obtained while falling can determine the force of the impact. Smart speaker technology is helping to improve the health status and quality of life for aging people by delivering services, including medicine reminders and communication to the caregiver or the family member. Our system utilizes a smart speaker-based voice assistant and IoT technology to verify whether the user has fallen by asking from a smart speaker. The state machine is used to directly operate the wearable device. Our system also provides portal access for health data exploration. The current posture of the user can be monitored in real-time via mobile devices or internet web pages. Our fall detection system provides real-time monitoring of a fall and can send an instant notification to the care provider or family member for immediate assistance. In addition, with the indoor positioning system, the historical data of behavioral patterns can be used to examine the long-term life habits and health status of the elderly. All obtained data can be provided for long-term systemic analysis as the basis for medical evaluation and management or provide a personalized fall prevention strategy.

## Figures and Tables

**Table 1:** The technical specifications of the sensors used for fall detection.

Bosch Sensortec	BMX055 Accelerometer	BMX055 Gyroscope	BMX055 Magnetometer	BMP280 Barometer
Operating range	±4 g	±1000 °/s	±1300 µT(x,y) ±2500 µT(z)	300 to 1100 hPa
Sensitivity	512 LSB/g	32.8 LSB/°/sec	3.3 LSB/µT	6 LSB/Pa
Accuracy	-	-	±2.5 degree	±1 hPa/±1 m/±1 °C
Interface type	I2C, SPI	I2C, SPI	I2C, SPI	I2C, SPI
Operating supply voltage (V)	1.2 to 3.6	1.2 to 3.6	1.2 to 3.6	1.71 to 3.6
Resolution (bit)	12	16	16	16 to 20
Sample rate (Hz)	100	100	100	100
Current consumption (mA)	0.13	5	0.8/4.9	0.0042
Operation case	All	Walking and falling (standing up and sitting down)	Walking and falling (standing up and sitting down)	Walking
Using time (h)	24	2	2	2
Power consumption (mAh)	3.12	10	1.6/9.8	0.0084

**Table 2:** The classification metrics (sensitivity, specificity, accuracy) of the three types of fall detection in the experiment.

Category	A	B	C
Type of fall	A fall while sitting down	A fall while standing	A fall while standing up
Sensitivity	94%	96%	95%
Specificity	95%	97%	95%
Accuracy	95%	96%	95%

**Table 3:** A comparison of wearable-based fall-related recognition systems in the referenced studies.

Ref	Sample Size (Persons)	Wearable Sensor (Type, Number, Location)	Methodology	Result
59	3	1 accelerometer	Quaternion algorithm using sum acceleration and rotation angle data	Sensitivity: 97.1%, Specificity: 98.3%. Accuracy: N/A
		Waist		
60	15	1 accelerometer 1 gyroscope 1 magnetometer	An algorithm based on acceleration and the angle of yaw, pitch, and roll, which is run on a smartphone	Sensitivity: 100% Specificity: 91.1% (shoulder) 100% (waist) 78.5% (foot) Accuracy: 100%
		Shoulder Waist Foot		
Our work	12	1 accelerometer, 1 gyroscope 1 magnetometer	Gradient descent algorithm based on the vector sum of acceleration and barometer record	Sensitivity: 94–96% Specificity: 95–97% Accuracy: 95–96%

**Table 4:** A comparison of existing commercial solutions for fall detection and real-time alerting with our proposed system.

	Sense4CareSTAT-ON	Lifeline AutoAlert	Apple Watch (Ultra 49 mm)	This Work
Detection sensor	three-axis accelerometer	three-axis accelerometer/ barometer	nine-axis accelerometer/barometer	nine-axis accelerometer/barometer
Sampling rate (Hz)	40	N/A	N/A	100
Size (mm)/ weight (g)	90 × 62.5 × 21.2/ 86	90 × 45 × 18/ 56	49 × 44 × 14.4/ 61	34 × 34 × 15/ 15
Battery (mAh)	1200	2000	542	150
Average current (mA)	4.1 ± 4.2	N/A	N/A	4.5 ±1.2
Battery life	8 h/day for 7 days = 56 h	5 days	Battery went from 100% down to 18% during 12 h	Continuous for 24 h
Installation location	Waist	Around the neck	Wrist	Chest
Fixation method	A belt	Pendants	A strap	Adhesives and necklace
Fall detection	Yes	Yes	Yes	Yes
Direction of the fall	No	No	No	Yes
Force of the fall	No	No	No	Yes
Posture before the fall	No	No	No	Yes
Movement and posture	For Parkinson’s disease	No	No	Yes
Combination with a smart speaker	No	Yes	Medical Guardian Mini Guardian (two-way speaker function, optional)	Yes
Combination with a physiological wristband	No	No	Has a physiological wristband function	Yes

## Data Availability

Not applicable.
